# Potential Alternative to Nitrite in Roasted Lamb for Sensory Attributes: Atmospheric Nonthermal Plasma Treatment

**DOI:** 10.3390/foods10061234

**Published:** 2021-05-28

**Authors:** Ruixia Chen, Dequan Zhang, Huan Liu, Zhenyu Wang, Teng Hui

**Affiliations:** 1 Institute of Food Science and Technology, Chinese Academy of Agricultural Sciences, Beijing 100193, China; cchenruixia@163.com (R.C.); dequan_zhang0118@126.com (D.Z.); sd_lh1990@126.com (H.L.); wangzhenyu@caas.cn (Z.W.); 2 Key Laboratory of Agro-Products Processing, Ministry of Agriculture and Rural Affairs, Beijing 100193, China

**Keywords:** roasted lamb, atmospheric nonthermal plasma, nitrite, sensory attribute, volatile compound

## Abstract

Reducing or replacing sodium nitrite without compromising the sensory attributes of meat products has always been a focus of the meat industry. In this study, five treatments, CT (without nitrite and plasma treatment), NT (with nitrite treatment), PT15, PT30, and PT45 (without nitrite and with plasma treatment for 15, 30, and 45 min, respectively), were designed to investigate the effect of atmospheric nonthermal plasma treatment replacing nitrite on the sensory attributes of roasted lamb. Results showed that PT45 decreased the residual nitrite of roasted lamb by 30% compared with NT, and nitrite was not detected in the PT15 and PT30 samples. The inhibition effect of plasma treatment on the lipid oxidation reached values from 86.69% to 89.89% compared with NT. Compared with CT, the redness of plasma-treated samples was increased by 9.30% to 31.40%, and the redness of NT samples was increased by 30.87%. In addition, the volatile compounds (OAVs > 1) of the PT30 sample were higher than those of the NT sample. The overall sensory score of the PT30 sample was higher than that of the CT sample and was similar to that of the NT samples. In conclusion, the sensory attributes of roasted lamb were enhanced by plasma treatment, and the 30 min plasma treatment is recommended.

## 1. Introduction

Roasted meat, with its attractive colour, tenderness, and unique flavour, is popular with consumers worldwide. It is usually made from raw meat cured with salt and other seasonings and then roasted with charcoal or electric heat. During the curing process, nitrite is usually applied to improve the colour, texture, and flavour of meat products; it also inhibits lipid oxidation, protein oxidation, and the growth of aerobic and anaerobic microorganisms [1,2]. However, during the thermal processing of meat products, nitrite and nitrate in meat are metabolized to nitrogen oxides, which in turn react with secondary amines to form carcinogenic *N*-nitrosamines [3,4,5].

For food safety concerns, the substitution of natural nitrite or nitrate for the meat industry has been widely studied [2,6,7]. Ozaki et al. [8] reported that radish and beetroot powders (0.5% and 1%) with *Staphylococcus carnosus* could be effectively used to replace nitrite in fermented sausages due to their high nitrate content. However, the content of nitrate in plants is easily affected by the external environment, such as temperature, season, soil, and storage conditions, and variety [1,7]. In addition, the excessive addition of some plant powder, such as cherry powder, can affect the taste and flavour of processed meat products, such as pork patties [2,9]. Therefore, it is necessary to find an effective replacement for nitrite/nitrate or new technology in the industry to achieve the multiple functions that nitrite plays during curing.

Nonthermal plasma, the fourth state of matter in the universe beside solid, liquid, and gas [10], is generated by an electric discharge in a gas at atmospheric or low-pressure conditions and contains free radicals, atoms, ions, particles, and molecules [11]. Recently, it has been reported that the reactive nitrogen species in atmospheric nonthermal plasma can react with water molecules and generate nitrite in liquid, brine, or meat batter [12,13,14]. Consequently, atmospheric nonthermal plasma is gradually being applied to replace nitrite in meat products, such as emulsion-type sausage, ground ham, and pork jerky [15,16,17,18]. Jung et al. [15] observed that the total aerobic bacteria, colour, peroxide value, and sensory attributes of emulsion-type sausages cured with plasma-treated water were similar to those of sausages cured with sodium nitrite. Another study reported that the degree of lipid and protein oxidation and sensory evaluation of canned ground hams cured with plasma had no significant differences with those cured by nitrite or celery powder [16]. Yong et al. [17] also found that the colour and residual nitrite content of pork jerky increased with increasing atmospheric pressure plasma treatment time, while the degree of lipid oxidation and pathogen count decreased. In addition, it was demonstrated that ground hams treated by remote plasma had a similar colour and lipid oxidation with those cured using nitrite, and the residual nitrite decreased rapidly during storage [18]. Based on the previous studies, atmospheric nonthermal plasma could be a potential method to replace sodium nitrite in meat product processing, while ensuring the sensory attributes of meat products.

Therefore, the aim of this study was to evaluate the effect of atmospheric nonthermal plasma treatments (15, 30, and 45 min) on the sensory attribute of roasted lamb compared with sodium nitrite treatment. The feasibility of replacing nitrite with plasma treatment was studied by measuring the residual nitrite content, protein and lipid oxidation, colour, shear force value, volatile compounds, and sensory score of roasted lamb. Accordingly, this is expected to provide theoretical support for the application of atmospheric nonthermal plasma treatment in the meat industry to produce nitrite-free or low-nitrite meat products.

## 2. Materials and Methods

### 2.1. Reagents and Materials

Sodium dodecyl sulphate (SDS) was purchased from Beijing Biodee Biotechnology Co., Ltd. (Beijing, China). Hydrochloric acid (HCl) and trichloroacetic acid (TCA) were purchased from Sinopharm Chemical Reagent Beijing Co., Ltd. (Beijing, China). The BCA protein assay kit was purchased from Thermo Fisher Technology Co., Ltd. (Shanghai, China). 2-Amino-2-(hydroxymethyl)-1,3-propanediol (Tris) and 5,5-dithiobis (2-nitrobenzoic acid) (DTNB) were obtained from Sigma-Aldrich Co., LLC. (Shanghai, China). Thiobarbituric acid (TBA) was bought from Yuanye Bio-Technology Co., Ltd. (Shanghai, China). 1,1,3,3-tetraethoxypropane (MDA) was purchased from Aladdin Biochemical Technology Co., Ltd. (Shanghai, China). *n*-Alkane (C_7_–C_40_, purity ≥ 97%) and 2-methyl-3-heptanone with a purity of 99% were purchased from o2si Smart Solutions (Shanghai, China) and Dr. Ehrenstorfer (Beijing, China), respectively. Onion and egg were obtained from the local market (Beijing, China), and table salt was obtained from China Salt Industry Co., Ltd. (Shanghai, China).

### 2.2. Roasted Lamb Processing

Twenty-five raw *silversides* from the left side of small male fat-tail sheep (same genetic background) were purchased from Grassland Hongbao Food Co., Ltd. (Inner Mongolia, China). The sheep were fed in confinement with a commercial diet (same feeding system) and, at 24.90 ± 0.55 kg live weight (approximately 7 months old), were stunned, slaughtered, and exsanguinated at a local slaughterhouse on the batch. Twenty-four hours post-mortem at 95% humidity and 4 °C, the ultimate pH in the *silversides* was 5.65 ± 0.03, and these *silversides* were then frozen at −30 °C. All *silversides* were vacuum packed and transported to our lab by cold-chain logistics and stored at −30 °C until used. The 25 *silversides* with the same genetic background and the same feeding system were consistent in biological information. The 25 *silversides* were randomly divided into five groups (five *silversides* in each group): CT (without sodium nitrite and plasma treatment), NT (with nitrite treatment), PT15 (without nitrite and with 15 min plasma treatment), PT30 (without nitrite and with 30 min plasma treatment), and PT45 (without nitrite and with 45 min plasma treatment). Each *silverside* was cut into 15 cubes of 3 cm × 3 cm × 1.5 cm (75 cubes in each group). Subsequently, each group was equally divided into three batches (25 cubes in each batch), in which the cubes from each batch represent one replicate sample, and the cubes from those three batches were cured independently: a total of 3 replicates were performed. The cubes in each batch from the five groups were cured according to Table 1. Briefly, the cubes from the first group were cured with 1% salt, 10% onion, and 3.5% egg white (without sodium nitrite and plasma, CT). The second group was cured with 1% salt, 10% onion, 3.5% egg white, and 0.005% sodium nitrite (NT). Another three groups were cured with 1% salt, 10% onion, and 3.5% egg white and subsequently treated by plasma for 15 min, 30 min, and 45 min, respectively (PT15, PT30, and PT45). These additives (salt, onion, egg white, and sodium nitrite) were added according to the weight of the meat, and curing was carried out at 4 ± 1 °C for 2 h. The atmospheric nonthermal plasma device was equipped with a dielectric-barrier discharge plasma system (DBD, SY-JXW-02, Suzhou FYB Agricultural Science and Technology Co., Ltd., Jiangsu, China), and it included a chamber for placing cured samples and a tube for transferring and cooling plasma gas. In this study, the plasma was discharged at a power of 40 W and a frequency of 10 kHz, and the flow rate of ambient air used as the process gas was 20 L/min. In addition, during plasma treatment, the temperature of the chamber was 0–5 °C.

After curing and plasma treatment, all the samples were roasted using an electric roasting oven (SJD-305-16, Xingguanyang Technology Co., Ltd., Wuxi, China). The temperature of the electric roasting oven was about 400–450 °C and lasted for 7 min, and the sample was turned over once per minute. For each batch in the same treatment group, 12 roasted cubes were immediately used for sensory evaluation after roasting, 4 roasted cubes were used for the detection of residual sodium nitrite, thiobarbituric acid value (TBARS value), and sulfhydryl content; 6 roasted cubes were used for the measurement of colour and shear force value; and 3 roasted cubes were used for the analysis of volatile compounds.

### 2.3. Residual Nitrite Contents in Roasted Lamb

The nitrite content in roasted lamb was measured using a nitrite detector (CSY-SY, FenXi Instruments Manufacturing Co., Ltd., Shenzhen, China) following the hydrochloride naphthodiamide method described by AOAC [19].

### 2.4. Determination of Sulfhydryl Content

Protein sulfhydryl content was measured using the process described by Bao et al. [20] with some modification. Briefly, 0.5 g of meat was homogenized with 12.5 mL of 5% (*w*/*v*) SDS in 0.1 mol/L Tris-HCl (pH 8.0) for 30 s using a homogenizer (Ultra-Turrax Disperser S25, IKA-Werke GmbH & Co. KG, Stafen, Germany) in an ice bath condition. The blended sample was heated in an 80 °C water bath for 30 min and cooled to ambient temperature. Next, the sample was filtered through Whatman NO.40 paper to collect the filtrate. The protein concentration of the filtrate was measured using the BCA Protein Quantitation Kit. A 0.5 mL amount of filtrate was mixed with 2 mL of 0.1 mol/L Tris-HCl (pH 8.0) and 0.5 mL of 10 mmol/L DTNB in 0.1 mmol/L Tris-HCl (pH 8.0) and incubated at ambient temperature for 30 min in the dark. The absorbance was determined at 412 nm against a blank sample containing 0.5 mL of 5% SDS, 2 mL of 0.1 mol/L Tris-HCl (pH 8.0), and 0.5 mL of 10 mmol/L DTNB in 0.1 mmol/L Tris-HCl (pH 8.0). The sulfhydryl content was calculated according to the following equation:

(1) $$c=\frac{A-A_{\mathrm{blank}}\times 10^{9}\times 6}{\varepsilon \;\times \;b\;\times c_{\mathrm{protein}}\;}$$

where *c* and *c*_protein_ are the sulfhydryl content (nmol/mg protein) and protein concentration (μg/mL) of the sample, respectively; *A* and *A*_blank_ are the absorbance of the sample and the blank sample reacting with DTNB at 412 nm, respectively; ε is the molar absorption coefficient (13,600 L/(mol·cm)); and b is the optical path (0.5 cm).

### 2.5. Determination of Thiobarbituric Acid Value (TBARS)

The TBARS value in the roasted lamb was determined by the method reported by Oz et al. [21] with some modifications. One gram of minced sample was mixed with 6 mL of 5% TCA in a 10 mL polypropylene tube and was homogenized for 30 s using a homogenizer (Ultra-Turrax Disperser S25, IKA-Werke GmbH & Co. KG, Stafen, Germany) in an ice bath condition. The blended sample was then centrifuged for 5 min (2000× *g*, 4 °C) and filtered using Whatman NO.1 paper filter. Next, 2 mL of filtrate and 2 mL of 0.02 mol/L TBA solutions were mixed in a 10 mL polypropylene tube. The solution was heated in a 95 °C water bath for 40 min and cooled to an ambient temperature. The absorbance was measured at 532 nm against a blank sample containing 2 mL of a 5% TCA solution and 2 mL of 0.02 mol/L TBA solutions. The TBARS value was calculated by the standard curve of the MDA solution, and the results were expressed as mg of MDA per kg of meat (mg MDA/kg meat).

### 2.6. Instrumental Colour

Objective colour (CIE lightness-L^*^, redness-a^*^, and yellowness-b^*^) on the surface of roasted lamb was measured using a Chromameter (CR600d1, Konica Minolta, Inc., Tokyo, Japan) with illuminant D_65_, a 10-degree standard observer, and an 8 mm aperture size. The colour measurement was performed perpendicular to three different locations per roasted lamb, and the results were analysed using Spectra Magic Software (Spectra Magic NX Ver. 2.8, Konica Minolta, Japan). Prior to measurement, the instrument was calibrated using a standard white plate, and the roasted lamb was cooled to room temperature.

### 2.7. Determination of Shear Force Value

The shear force value of the roasted lamb was determined using a muscle tenderness meter (C-LM4, Northeast Agricultural University, Harbin, China), according to the method reported by Xiao et al. [22] and with some modification. Shear force value was determined after the roasted lamb was cooled to room temperature and cut into cuboids (2 cm × 1 cm × 1 cm), and the shear velocity was 5 mm/s. 

### 2.8. Determination of Volatile Compounds

Volatile compounds in the roasted lamb were extracted by the solid-phase microextraction (SPME) method and subsequently analysed by gas chromatography coupled to mass spectrometry (GCMS-QP 2010 Plus, Shimadzu, Kyoto, Japan) according to the literature reported by Liu et al. [23]. The extraction conditions were as follows: 2.0 g of minced sample were placed in a 20 mL headspace vial, and 1.5 µL of internal standard (2-methyl-3-heptanone, 1.68 µg/µL in methanol) was added, the vial sealed with a PTEE-silicon stopper. The vial was put in a temperature-controlled water bath at 50 °C for 20 min, and the volatile compound was extracted by an SPME fibre coated with carbon-polydimethylsiloxane (DVB/PDMS, 65 µm). After extraction, the SPME fibre was immediately transferred to the GC injection port and desorbed at 200 °C for 2 min with splitless mode. Volatile compounds were separated using a DB-WAX capillary column (30 m × 0.25 µm × 0.25 µm, Aglient Technologies, Santa Clara, CA, USA), and the carrier gas was helium at a constant flow rate of 1.0 mL/min. The temperature program was set as follows: the oven temperature was kept at 40 °C for 3 min, increased to 120 °C at 5 °C/min, and subsequently increased to 200 °C at 10 °C/min and held for 13 min. The temperature of the ion source and the GC-MS transfer line was 200 °C and 250 °C, respectively. The mass spectrometer was operated in the electron impact mode with an electron energy of 70 eV and collected data at a range of m/z 35-500 in full-scan mode. 

Volatile compounds were confirmed by matching their mass spectra with those involved in the NTIS 11 or NTIS 11s mass spectrometry library and comparing their line retention indexes (LRI) with those reported in the NIST Chemistry WebBook-SRD69 [24]. Volatile compounds were semiquantitated based on 2-methyl-3-heptanone. The concentrations of the volatile compounds were calculated according to the ratio between peak area and the concentration of 2-methyl-3-heptanone. The contribution of volatile compounds to the flavour of roasted lamb were evaluated by odour activity values (OAVs), which were calculated as the ratio of the compound concentration to the perception threshold described in the literature [23] and the book of compilations of odour threshold in air, water, and other media [25]. The major contributors of the roasted meat flavour were generally considered to be volatile compounds with OAVs > 1 [26]. 

### 2.9. Sensory Evaluation 

Sensory evaluation was carried out by 12 trained panellists (three males and nine females) recruited from our laboratory by following the protocols of Xiao et al. [22]. Prior to sensory evaluation, all panellists were selected after preliminary screening sessions and after three training sessions on the descriptors and methodologies directed at roasted lamb [22]. After a group discussion, six sensory attributes (lamb odour, roasted flavour, colour, tenderness, juiciness, and overall acceptance) were selected to access the organoleptic property of roasted lamb. The performance of the panellists was estimated by Generalized Procrustes analysis (GPA). A low residual variance (<2), scaling factors (near 1), and overlapped positions of variables and products obtained by the 12 panellists from the GPA indicated that high levels of group homogeneity were procured [27]. After the group panellists reached high levels of agreement, three sessions were performed, and 6 roasted lamb samples (1 dummy sample + 5 test samples) were evaluated at each session. Each sample was assessed in triplicate by each panellist. Test samples labelled with 3-digit numeric codes were presented to panellists in a random order. During the evaluation, each panellist was requested to drink water between each sample to avoid sample interaction. At the beginning of the session, the lamb odour of roasted lamb samples was evaluated by a panellist, and the panellist was then asked to evaluate the colour before tasting. The sensory evaluation was based on a 9-point linear scale to determine the colour of the surface skin (9 = red; 1= dark), lamb odour, roasted flavour (9 = intense; 1 = mild), tenderness, juiciness (9 = intense; 1 = mild), and overall acceptance (9 = high; 1 = low). The average of the sensory scores obtained by the twelve panellists of the 6 sensory attributes for each roasted sample was collected for further analysis.

### 2.10. Statistical Analysis

The results were expressed as the mean ± standard deviation. The statistical analysis of the residual nitrite content, sulfhydryl content, TBARS value, colour, shear force value, volatile compound content, and sensory scores in all treatment samples were analysed by one-way ANOVA followed by least significant differences (LSD) multiple comparison tests (*p* < 0.05) using the SPSS statistical software (SPSS Ver. 17, SPSS Inc., Chicago, IL, USA). To distinguish the differences in sensory scores between the roasted lamb treated with five treatments (CT, NT, PT15, PT30, and PT45), an orthogonal partial least squares discrimination analysis (OPLS-DA) was performed with SIMCA software (Version 14.1, Sartorius AG, Göttingen, Germany). The five treatments were used as Y-variables, and the sensory scores were used as X-variables. A bioplot of OPLS-DA displayed the differences in sensory scores of the roasted lamb with different treatments. The orthogonal partial least squares discrimination analysis (OPLS-DA) of volatile compounds (OAVs > 1) in roasted lamb was also performed with SIMCA software (Version 14.1, Sartorius AG, Germany). The five treatments were selected as observations, and the volatile compounds (OAVs > 1) were selected as the variables. The OPLS-DA score plot was used to distinguish the difference between the five treatments.

## 3. Results and Discussion

### 3.1. Nitrite Content in Roasted Lamb

The residual nitrite content in roasted lamb is presented in Table 2. Nitrite was not detected in samples treated with plasma for 15 min (PT15) and 30 min (PT30). However, the residual nitrite content of the sample prepared with 45 min plasma was 1.88 mg/kg nitrite, which showed a rapid increase with the increase of treatment time from 30 to 45 min. Yong et al. [17] reported that the nitrite content of the plasma-treated brine increased with the increase of treatment time from 0 to 60 min. This phenomenon was caused by the nitric oxide and other nitrogen oxides in plasma that can react with water molecules to generate nitrite [28,29]. Furthermore, the residual nitrite content of roasted lamb prepared with 45 min plasma treatment was 30% lower than nitrite treatment. 

### 3.2. Protein and Lipid Oxidation

**Figure 1:**
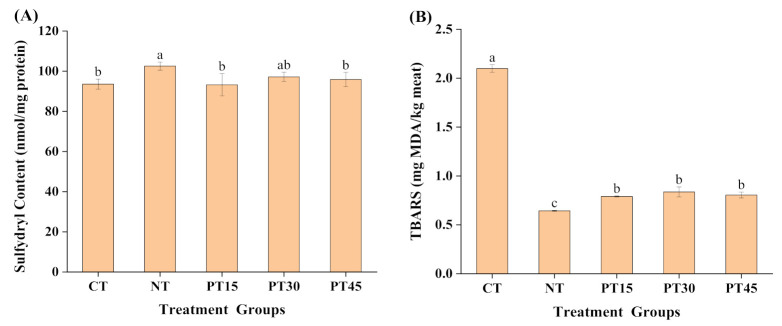
Changes in the content of sulfhydryl (**A**) and TBARS value (**B**) of roasted lamb with different treatments. ^a^^, b, c^ Mean values with different letters are significantly different (*p* < 0.05). Control group (CT): without sodium nitrite and plasma treatment; NT: with 0.005% sodium nitrite; PT15: with plasma treatment for 15 min; PT30: with plasma treatment for 30 min; PT45: with plasma treatment for 45 min.

The sulfhydryl content was measured to evaluate the degree of protein oxidation in the samples, and the results are shown in Figure 1A. Compared with the control group (CT), the sulfhydryl content of samples prepared with nitrite (NT) significantly (*p* < 0.05) increased. This result indicated that the addition of sodium nitrite can inhibit the degree of protein oxidation in roasted lamb. This might be explained by the antioxidant activity of nitric oxide generated by the decomposition of nitrite during the processing of roasted lamb [2]. In addition, compared with the control sample, the sulfhydryl content of samples prepared with 30 min (PT30) and 45 min (PT45) plasma treatment increased by 3.85% and 2.45%, respectively, although there were not statistically significant (*p* > 0.05) differences. The results clearly showed that the plasma treatment did not aggravate the protein oxidation of roasted lamb in this study. In contrast, Huang et al. [30] found that the plasma treatment for 60 s could exacerbate the protein oxidation of modified-package pork by the reactive oxygen species interacting with the protein. This difference may be caused by the different processing parameters, packing methods, and the meat types.

Malondialdehyde (MDA), a secondary product of lipid oxidation, is usually determined by the TBARS analysis to evaluate the degree of lipid oxidation. The TBARS values are shown in Figure 1B. NT, PT15, PT30, and PT45 reduced the TBARS value by 69.35%, 62.34%, 60.12%, and 61.66% compared with CT, respectively. The result demonstrated that the degree of lipid oxidation in roasted lamb was inhibited by sodium nitrite and plasma treatment, and the inhibition effect of plasma treatment on lipid oxidation reached values from 86.69% to 89.89% compared with the sodium nitrite treatment. Previous studies have found that the nitrite can inhibit lipid oxidation in cured meat products such as ground ham [4,18,28]. It is well known that the initiation of lipid oxidation is inhibited by the reaction of nitrite and oxygen species, and the lipid oxidation is terminated by the reaction of nitric oxide and lipid peroxyl radicals [28]. Generally, the reactive oxygen species (particularly the free radicals) from plasma can interact with lipids in the plasma-treated product and initiate the oxidation [31]. It has been reported that the plasma treatment increased the TBARS value in modified-package pork by promoting lipid oxidation [30]. The difference in the result from that of our study can be explained by the fact that the oxygen in the package promoted the formation of ozone and accelerated lipid oxidation. However, some studies have demonstrated that plasma treatment had no effect on the lipid oxidation of meat batter or canned ground ham [16,32]. The differences in these results from our study could be due to the difference in the meat product type and the composition of gas used for generating plasma in these studies. Kim et al. [33] reported that the TBARS value of bacon treated by helium-based plasma showed no significant difference from that of bacon treated by helium/oxygen-mixture-based plasma. However, the atmosphere was used to generate plasma in our study. Previous studies also reported that the type and content of reactive species-initiated lipid oxidation can be affected by the composition of gas in plasma [11,31]. 

### 3.3. Colour and Shear Force Value

**Figure 2:**
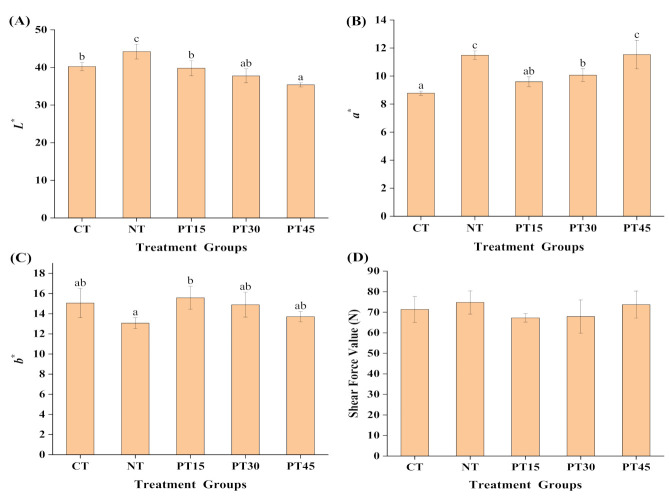
Changes in colour (L^*^-(**A**), a^*^-(**B**), b^*^-(**C**)) and shear force value (**D**) of roasted lamb with different treatments. ^a^^, b, c^ Mean values with different letters are significantly different (*p* < 0.05). Control group (CT): without sodium nitrite and plasma treatment; NT: with 0.005% nitrite sodium; PT15: with plasma treatment for 15 min; PT30: with plasma treatment for 30 min; PT45: with plasma treatment for 45 min.

As shown in Figure 2A, compared with CT, the L^*^ value (lightness) of the PT15 and PT30 samples had no significant (*p* > 0.05) differences, but it was significantly (*p* < 0.05) higher than PT45, while the L^*^ value of NT significantly (*p* < 0.05) increased. In addition, there were no significant (*p* > 0.05) differences between the plasma treatment group and the control group with respect to the value of yellowness (b^*^) presented in Figure 2C, but the b^*^ value of NT samples decreased. For the value of redness (a^*^), NT samples increased 30.87% compared with the CT groups (Figure 2B) and increased the roasted lamb’s redness, which was also an important reason for adding nitrite in the process of roasted lamb. Nitrite can decompose into nitric oxide (NO), after which NO reacts with myoglobin to form pink nitrosomyoglobin during the heating process in meat products [34]. Interestingly, the a^*^ value of the roasted lamb increased as plasma treatment time increased. The redness (a^*^ value) of the roasted lamb prepared by plasma treatment for 15 min, 30 min, and 45 min increased by 9.30%, 14.65%, and 31.40% compared with the control treatment, respectively. Meanwhile, the redness of the PT45 samples was similar to that of the NT samples. Similarly, Yong et al. [17] also observed that the a^*^ value of the 60 min plasma-treated pork jerky increased significantly (*p* < 0.05), while the a^*^ value of the 40 min plasma-treated jerky was comparable with the sodium nitrite-treated ones. It has also been reported that the a^*^ value and nitrite content of ground ham and meat batter also increased with the atmospheric nonthermal plasma treatment time [18,32]. 

As shown in Figure 2D, the shear force value of the samples showed no significant (*p* > 0.05) differences among all treatment groups, indicating that the tenderness of the samples was not influenced by the plasma treatment. A previous study also reported that the atmospheric pressure plasma treatment had no influence on the textural properties of the canned ground hams [16]. The results in this study clearly showed that plasma treatment can enhance the redness of roasted lamb while having no influence on its tenderness.

### 3.4. Volatile Compounds

In this study, a total of 35 volatile compounds were detected in the five groups of roasted lamb by GC-MS and were classified as follows: fifteen aldehydes, eight alcohols, three ketones, four pyrazines, two acids, one furan, one ester, and one pyrrole (Table 3). 

**Figure 3:**
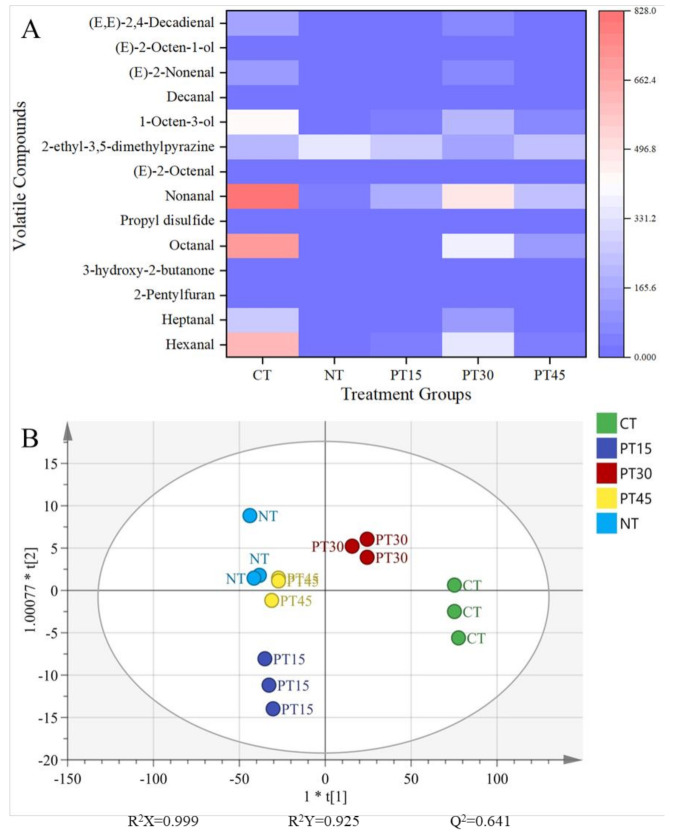
A heatmap (**A**) displaying the OAVs of volatile compounds (OAVs > 1) of roasted lamb, and an OPLS-DA score plot (**B**) displaying the comparison of volatile compounds (OAVs > 1) in roasted lamb with different treatments. Control group (CT): without sodium nitrite and plasma treatment; NT: with 0.005% sodium nitrite; PT15: with plasma treatment for 15 min; PT30: with plasma treatment for 30 min; PT45: with plasma treatment for 45 min.

Figure 3A shows that in the CT samples, the volatile compounds with OAVs > 1 included eight kinds of aldehydes (hexanal, heptanal, octanal, nonanal, (E)-2-octenal, (E)-2-nonenal, decanal, and (E, E)-2,4-decadienal), three alcohols (1-octen-3-ol, (E)-2-octen-1-ol, and propyl disulfide), 2-ethyl-3,5-dimethylpyrazine, and 3-hydroxy-2-butanone. These 13 volatile compounds may be the main contributors for the flavour of the CT samples. As shown in Table 3, the contents of these 13 volatile compounds were affected by the nitrite and plasma treatment. In terms of nitrite treatment, the sample contained only two kinds of aldehydes (octanal and nonanal), three alcohols (1-octen-3-ol, (E)-2-octen-1-ol, and propyl disulfide), 2-ethyl-3,5-dimethylpyrazine, and 3-hydroxy-2-butanone. The PT15 sample included three aldehydes (hexanal, nonanal and decanal), two alcohols (1-octen-3-ol and (E)-2-octen-1-ol), 2-ethyl-3,5-dimethylpyrazine, and 3-hydroxy-2-butanone. The PT30 sample contained various volatile compounds (OAVs > 1) that were in the CT sample, except (E,E)-2,4-decadienal. The PT45 sample included four aldehydes (hexanal, octanal, nonanal, and decanal), three alcohols (1-octen-3-ol, (E)-2-octen-1-ol, and propyl disulfide), 3-hydroxy-2-butanone, and 2-ethyl-3,5-dimethylpyrazine. Similarly, Luo et al. [14] found that the formation of volatile compounds in dried pork loin was affected when plasma-treated water was used in the marinating process.

Compared with the CT samples, the content of hexanal significantly (*p* < 0.05) decreased in the plasma-treated samples, and none was detected in the nitrite-treated samples. This result is consistent with the TBARS result, since hexanal is usually produced by linoleic acid oxidation [35]. In addition, it has been reported that the formation of hexanal can be inhibited by nitrite curing in meat products such as dry fermented sausages [35,36]. Similarly, the contents of nonanal in the PT and NT samples were significantly (*p* < 0.05) lower than those of the CT samples. Luo et al. [14] also reported that the nonanal concentration of Chinese dried pork loin marinated with untreated brine was higher than that of pork loin marinated with plasma-treated water. In addition, compared with CT samples, the content of heptanal, octanal, (E)-2-octenal, decanal, and (E)-2-nonenal decreased by 56.09%, 47.94%, 57.24%, 11.19%, and 49.25%, respectively, in the PT30 samples, while these aldehydes were not detected in the NT, PT15, and PT45 samples. (E,E)-2,4-Decadienal was not detected in the PT and NT samples. As we know, the formation of aldehyde compounds is related to the protein degradation and lipid oxidation during the processing of roasted meat [35]. The decrease of such aldehydes in roasted lamb treated with plasma and nitrite might be the result of the decrease in lipid oxidation during processing. The contents of hexanal, nonanal, heptanal, octanal, E-2-octenal, decanal, and(E)-2-nonenal in the PT samples were higher than those of the NT samples. Perea-Sanz et al. [37] also found that total aldehydes in fermented sausages with 80 mg/kg nitrite and 95 mg/kg nitrate obviously decreased compared with those with 150 mg/kg nitrite and 178 mg/kg nitrate.

Compared with CT samples, the propyl disulfide content significantly (*p* < 0.05) increased in the PT30 and NT samples, while the contents of E-2-octen-1-ol, and 1-octen-3-ol significantly (*p* < 0.05) decreased. 1-Octen-3-ol, with a mushroom flavour, is usually generated from linoleic acid β-oxidation [14,37]. In this study, the inhibition of lipid oxidation caused by plasma treatment gave rise to a reduction of 1-octen-3-ol compared with the CT samples. Similarly, the content of 1-octen-3-ol of the PT samples was higher than that of the NT samples. The reason might be the fact that the inhibitory rate of lipid oxidation in the plasma treatment was significantly (*p* < 0.05) lower compared with the nitrite treatment. The content of 3-hydroxy-2-butanone in the CT sample was significantly (*p* < 0.05) higher than that of the NT, PT30, and PT45 treatments, but there was no significant (*p* > 0.05) difference in samples between CT and PT15 treatments. 3-Hydroxy-2-butanone is mainly derived from carbohydrate fermentation [37]. Yong et al. [17] showed that plasma treatment can inhibit the microbial growth in pork jerky during processing, so long-term plasma treatment in PT30 and PT45 might inhibit microbial growth related to the generation of 3-hydroxy-2-butanone. 2-Ethyl-3,5-dimethylpyrazine, as one of the pyrazine compounds, might be related to the roasted flavour in roasted lamb. There were no significant (*p* > 0.05) differences in the content of 2-ethyl-3,5-dimethylpyrazine in roasted lamb among all treatments. Luo et al. [14] also showed that pyrazine compounds in dried pork loins were not decreased after marinating them with plasma-treated water.

The score plot of orthogonal partial least squares discriminant analysis (OPLS-DA) is an effective discriminant way to separate the difference among experiment treatments [38]. The OPLS-DA of volatile compounds (OAVs > 1) in roasted lamb with different treatments was carried out to intuitively clarify the effect of plasma treatment on the flavour of roasted lamb. The result is shown in Figure 3B. The CT and PT30 groups were distributed in the positive *x*-axis direction, and the PT15, PT45, and NT groups were located in the negative *x*-axis direction. Obviously, the distance between the PT30 and CT groups was smaller in the score plot compared with other treatments, indicating that the PT30 treatment had the least effect on the profiles of volatile compounds (OAVs > 1). In other word, the kinds and contents of volatile compounds (OAVs > 1) in the PT30 samples were most similar to those in the CT samples, rather than those of the NT samples. 

### 3.5. Sensory Evaluation

**Figure 4:**
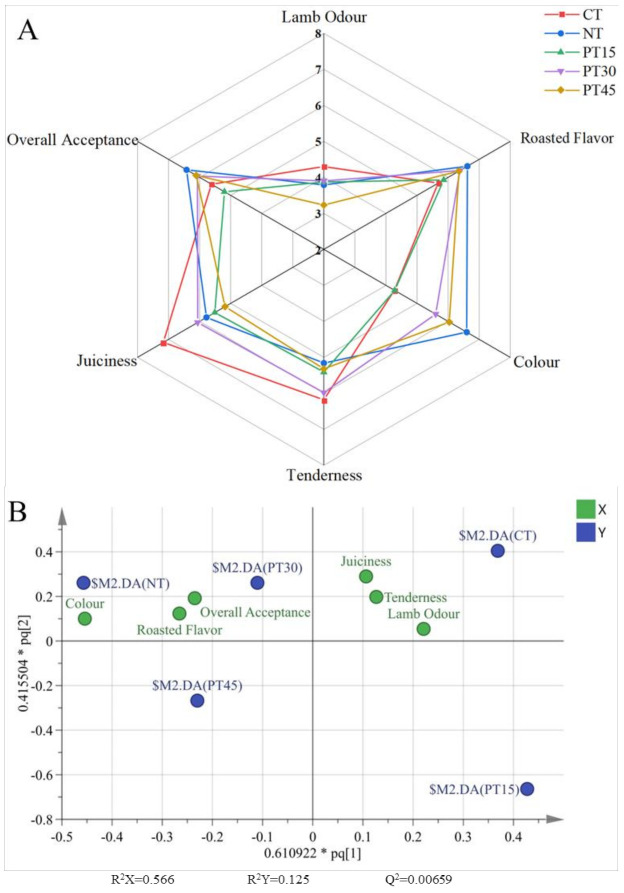
Radar (**A**) displaying the sensory scores of lamb odour, roasted flavour, colour, tenderness, juiciness, and overall acceptance in roasted lamb and OPLS-DA bioplot (**B**) displaying the comparison of sensory scores in roasted lamb with different treatments. Control group (CT): without sodium nitrite and plasma treatment; NT: with 0.005% sodium nitrite; PT15: with plasma treatment for 15 min; PT30: with plasma treatment for 30 min; PT45: with plasma treatment for 45 min.

The sensory scores of the lamb odour, roasted flavour, colour, tenderness, juiciness, and overall acceptance of the roasted lamb are shown in Figure 4A. The average sensory scores of tenderness and juiciness in the PT30 samples were 5.98 and 6.06 (Table A1), respectively, which were the closest to those of the CT samples and higher than those of the NT samples. The sensory scores of the roasted flavour in the PT30, PT45, and NT samples were higher than that of the CT sample. Table 3 shows that pyrazine compounds with a roasted aroma were found in all treatments, and the contents and kinds of pyrazines in the NT, PT30, and PT45 samples were higher than those of the CT samples, which contributed to a higher roasted flavour score in the NT, PT30, and PT45 samples compared with the CT samples. Hexanal and 1-octen-3-ol, derived from lipid oxidation, are responsible for rancid and oxidized flavours in meat products [39]. The contents of these two aldehydes were higher in the CT samples compared with those of the NT and PT samples, which might decrease the roasted flavour in the CT samples. Meanwhile, the lamb odour scores of the PT30 and PT45 samples were lower than the CT samples, which are closely related to the contents of short chain fatty acids such as 4-methyloctanoicacid, 4-4-methylnonanoicacid, and 4-ethyloctanoicacid [40]. Giannoglou et al. [41] reported that a 15 min plasma treatment with a 45-kHz frequency might decompose the fatty acids and then decrease their contents in sea bream fillets. Most likely, the plasma treatment for roasted lamb in this study reduces the contents of short chain fatty acids related to the lamb odour and thus partly leads to their higher overall acceptance compared with the CT samples. In addition, the colour scores of the PT30, PT45, and NT samples from the sensory evaluation were higher than those of the CT samples. The result of instrumental colour showed that the redness (a^*^) values of the NT, PT30, and PT45 samples were significantly (*p* < 0.05) higher than those of the CT samples, which might lead to higher colour scores in the PT30, PT45, and NT samples. 

The orthogonal partial least squares discrimination analysis (OPLS-DA) bioplot of the sensory score is shown in Figure 4B. In the OPLS-DA bioplot, the distance between the treatment group and the sensory attribute was smaller, which means that the roasted lamb in the treatment group has a higher sensory score. The length of the distances among the different treatment groups indicated the degree of difference of samples in those groups. The result showed that the NT samples had a higher colour score, and the PT30 samples had higher overall acceptance and roasted flavour scores compared with other treatments. In the bioplot, the shortest distance among all treatments was between the PT30 group and the NT group, which means the sensory attributes were similar between these two treatments. Therefore, the sensory attributes of roasted lamb were enhanced by the 30 min plasma treatment. 

## 4. Conclusions

The residual nitrite content of the PT45 samples was lower than that of the nitrite treatment samples and was not detected at all in the PT15 and PT30 samples. The inhibition effect of the plasma treatment on lipid oxidation reached 86.69% to 89.89% compared with nitrite treatment. Meanwhile, the redness of the samples increased as the time of the plasma treatment increased, and the redness of the PT45 samples was similar to that of the NT samples. In addition, the plasma treatment had no obvious effect on the tenderness of the roasted lamb. The kinds and contents of volatile compounds (OAVs > 1) in the PT30 samples were similar to those of the CT samples, and were higher than those of the NT samples. In addition, the overall sensory score of the PT 30 samples was higher than that of the CT samples and was similar to that of the NT samples. In summary, the sensory attributes of the roasted lamb were enhanced by the plasma treatment, and the 30 min plasma treatment had improved sensory attributes. Consequently, plasma treatment can be used as an alternative method to produce nitrite-free or low-nitrite roasted lamb without compromising the sensory attributes of the products. However, the microbial stability and security, e.g., oral toxicity and teratogenicity, of plasma-treated roasted lamb should be studied to provide strong theoretical support for the application of atmospheric nonplasma treatment in the meat processing industry.

## Figures and Tables

**Table 1:** Experimental design: salt, onion, egg white, and sodium nitrite (in percentage of meat weight) used for meat samples.

Treatment Groups ^a^	CT	NT	PT15	PT30	PT45
Salt	1%	1%	1%	1%	1%
Onion	10%	10%	10%	10%	10%
Egg white	3.5%	3.5%	3.5%	3.5%	3.5%
Nitrite sodium	-	0.005%	-	-	-
Plasma treatment time	-	-	15 min	30 min	45 min

^a^ Treatment groups: Control group (CT): without sodium nitrite and plasma treatment; NT: with 0.005% sodium nitrite; PT15: with plasma treatment for 15 min; PT30: with plasma treatment for 30 min; PT45: with plasma treatment for 45 min.

**Table 2:** Changes in nitrite content (mg/kg) in roasted lamb.

	CT	NT	PT15	PT30	PT45
Nitrite content (mg/kg)	ND	2.66 ± 0.82 ^a^	ND	ND	1.88 ± 0.59 ^b^

^a,b^ Mean values in the same row followed by different letters are significantly different (*p* < 0.05). ND: Not detected; Control group (CT): without sodium nitrite and plasma treatment; NT: with 0.005% sodium nitrite; PT15: with plasma treatment for 15 min; PT30: with plasma treatment for 30 min; PT45: with plasma treatment for 45 min.

**Table 3:** Changes in volatile compound content (ng/g) in roasted lamb.

	LRI ^a^		Identification ^d^	CT	NT	PT15	PT30	PT45
	Literature ^b^	Calculated ^c^						
Aldehydes								
Hexanal	1073	1077	MS + LRI	2773.56 ± 423.64 ^a^	ND	131.09 ± 41.18 ^c^	1572.25 ± 120.73 ^b^	224.03 ± 39.84 ^c^
Heptanal	1163	1180	MS + LRI	786.22 ± 270.82 ^a^	ND	ND	345.23 ± 2.18 ^b^	ND
Octanal	1275	1283	MS + LRI	484.85 ± 136.37 ^a^	20.36 ± 6.54 ^c^	ND	252.42 ± 20.73 ^b^	91.30 ± 7.52 ^c^
(E)-2-Heptenal	1334	1311	MS + LRI	17.25 ± 6.05	ND	ND	ND	ND
Nonanal	1385	1387	MS + LRI	826.55 ± 159.97 ^a^	47.40 ± 11.09 ^d^	192.82 ± 33.86 ^c^	483.55 ± 16.69 ^b^	232.68 ± 21.39 ^c^
(E)-2-Octenal	1416	1419	MS + LRI	38.28 ± 10.28 ^a^	ND	ND	16.37 ± 7.92 ^b^	ND
Decanal	1483	1492	MS + LRI	11.71 ± 0.33 ^a^	ND	1.95 ± 0.39 ^d^	10.40 ± 0.88 ^b^	4.39 ± 0.43 ^c^
Benzaldehyde	1508	1506	MS + LRI	43.30 ± 5.40 ^a^	28.75 ± 6.81 ^b^	31.55 ± 4.69 ^b^	36.15 ± 2.23 ^ab^	30.27 ± 2.28 ^b^
(E)-2-Nonenal	1535	1526	MS + LRI	28.57 ± 7.64 ^a^	ND	ND	14.50 ± 5.24 ^b^	ND
Dodecanal	1710	1704	MS + LRI	6.24 ± 1.23 ^a^	ND	ND	4.47 ± 0.87 ^b^	ND
2-Undecenal	1755	1745	MS + LRI	7.98 ± 2.23	ND	ND	ND	ND
(E,E)-2,4-Decadienal	1766	1757	MS + LRI	11.12 ± 2.80	ND	ND	ND	ND
Myristaldehyde	1931	1918	MS + LRI	8.77 ± 2.79 ^a^	ND	ND	4.27 ± 1.00 ^b^	ND
Pentadecanal	2042	2025	MS + LRI	11.86 ± 4.29 ^a^	ND	1.32 ± 0.70 ^b^	7.19 ± 3.14 ^a^	ND
Hexadecanal	2141	2132	MS + LRI	4.41 ± 0.34 ^a^	ND	2.36 ± 0.43 ^b^	4.22 ± 0.35 ^a^	1.90 ± 0.27 ^b^
**Alcohols**								
Pentanol	1252	1250	MS + LRI	177.44 ± 30.82 ^a^	ND	7.62 ± 1.74 ^c^	77.33 ± 12.57 ^b^	9.84 ± 1.83 ^c^
Hexanol	1344	1352	MS + LRI	45.99 ± 8.11 ^a^	ND	4.80 ± 0.72 ^c^	33.02 ± 10.66 ^b^	5.74 ± 1.46 ^c^
Propyl disulfide	1378	1369	MS + LRI	26.31 ± 4.54 ^b^	63.9 ± 18.74 ^a^	ND	46.30 ± 3.39 ^a^	4.29 ± 0.70 ^c^
1-Octen-3-ol	1430	1449	MS + LRI	433.01 ± 89.65 ^a^	15.58 ± 4.35 ^c^	42.73 ± 8.22 ^c^	202.97 ± 17.34 ^b^	57.32 ± 8.22 ^c^
Heptanol	1456	1454	MS + LRI	46.41 ± 8.66 ^a^	ND	ND	24.57 ± 2.51 ^b^	ND
2-Ethyl-1-hexanol	1484	1488	MS + LRI	ND	ND	ND	4.07 ± 0.41	ND
Octanol	1554	1556	MS + LRI	48.19 ± 13.49 ^a^	2.50 ± 0.74 ^c^	9.07 ± 1.38 ^c^	26.34 ± 2.17 ^b^	12.36 ± 0.97 ^c^
(E)-2-Octen-1-ol	1620	1613	MS + LRI	51.71 ± 11.01 ^a^	1.85 ± 0.51 ^c^	4.66 ± 0.68 ^c^	22.16 ± 2.96 ^b^	6.66 ± 0.72^c^
**K** **etones**								
3-Hydroxy-2-butanone	1280	1273	MS + LRI	115.51 ± 18.81 ^a^	56.61 ± 16.32 ^b^	110.85 ± 18.59 ^a^	59.86 ± 5.69 ^b^	48.70 ± 4.52 ^b^
2,3-Octanedione	1325	1321	MS + LRI	1373.31 ± 292.40 ^a^	ND	48.19 ± 18.95 ^c^	645.8 ± 45.51 ^b^	80.81 ± 20.84 ^c^
6-Methyl-5-heptene-2-one	1340	1328	MS + LRI	ND	ND	2.80 ± 0.09 ^b^	6.29 ± 0.74 ^a^	ND
**P** **yrazines**								
2,5-Dimethylpyrazine	1325	1314	MS + LRI	ND	22.27 ± 7.51 ^a^	ND	14.22 ± 2.72 ^b^	14.25 ± 1.53 ^b^
2-Methyl-6-ethylpyrazine	1393	1382	MS + LRI	ND	8.80 ± 5.27 ^a^	3.48 ± 0.23 ^b^	3.89 ± 0.74 ^b^	8.88 ± 1.48 ^a^
2,3,5-Trimethylpyrazine	1408	1395	MS + LRI	18.68 ± 4.23	18.89 ± 6.70	15.22 ± 2.26	12.87 ± 1.48	17.97 ± 2.42
2-Ethyl-3,5-dimethylpyrazine	1443	1438	MS + LRI	8.09 ± 4.37 ^ab^	13.40 ± 3.89 ^a^	10.15 ± 1.24 ^ab^	6.24 ± 0.36 ^b^	9.49 ± 1.56 ^ab^
**A** **cids**								
Butanoic acid	1625	1621	MS + LRI	ND	7.63 ± 2.33 ^b^	13.94 ± 2.52 ^a^	ND	11.73 ± 1.42 ^a^
Hexanoic acid	1850	1839	MS + LRI	45.58 ± 15.17 ^a^	5.41 ± 1.51 ^c^	10.42 ± 1.40 ^c^	25.97 ± 6.43 ^b^	12.05 ± 1.32 ^c^
**O** **thers**								
2-Pentylfuran	1235	1228	MS + LRI	103.49 ± 23.50 ^a^	ND	13.25 ± 3.65 ^c^	59.97 ± 7.54 ^b^	19.16 ± 1.07 ^c^
Butyrolactone	1615	1606	MS + LRI	4.74 ± 1.45 ^a^	3.96 ± 1.07 ^ab^	5.11 ± 0.59 ^a^	2.92 ± 0.11 ^b^	4.58 ± 0.52 ^ab^
2-Acetylpyrrole	1972	1960	MS + LRI	2.74 ± 0.59 ^a^	1.90 ± 0.60 ^b^	2.02 ± 0.22 ^ab^	2.32 ± 0.14 ^ab^	1.67 ± 0.17 ^b^

^a^^, b,^^c^ Mean values in the same row followed by different letters are significantly different (*p* < 0.05). ^a^ Linear retention index. ^b^ Reported date. ^c^ Calculated data based on n-alkanes (C_7_–C_40_). ^d^ Reliability of identification. MS: volatile compounds tentatively identified by comparing their mass spectra with those contained in the MS library; LRI: volatile compounds tentatively identified by comparing their LRI with those reported in the literature; ND: Not detected; Control group (CT): without sodium nitrite and plasma treatment; NT: with 0.005% sodium nitrite; PT15: with plasma treatment for 15 min; PT30: with plasma treatment for 30 min; PT45: with plasma treatment for 45 min.

## Data Availability

The data that support the findings of this study are available from corresponding author upon request.

## Appendix A

**Table A1:** The sensory score of lamb odour, roasted flavour, colour, tenderness, juiciness, and overall acceptance in roasted lamb.

	Colour	Lamb Odour	Tenderness	Juiciness	Roasted Flavour	Overall Acceptance
CT	4.27 ± 1.39 ^a^	4.30 ± 2.04 ^a^	6.18 ± 1.32 ^a^	7.17 ± 1.61 ^b^	5.68 ± 1.43 ^a^	5.62 ± 1.37 ^ab^
NT	6.60 ± 1.82 ^b^	3.79 ± 2.12 ^a^	5.16 ± 1.62 ^a^	5.78 ± 1.50 ^a^	6.62 ± 1.05 ^a^	6.42 ± 0.90 ^b^
PT15	4.28 ± 1.98 ^a^	3.87 ± 2.09 ^a^	5.41 ± 1.49 ^a^	5.51 ± 1.34 ^a^	5.86 ± 1.57 ^a^	5.20 ± 1.82 ^a^
PT30	5.60 ± 1.55 ^ab^	3.90 ± 1.87 ^a^	5.98 ± 1.63 ^a^	6.06 ± 1.60 ^ab^	6.38 ± 1.11 ^a^	6.07 ± 1.27 ^ab^
PT45	6.04 ± 1.05 ^b^	3.23 ± 1.71 ^a^	5.32 ± 1.21 ^a^	5.18 ± 1.77 ^a^	6.35 ± 1.31 ^a^	6.11 ± 0.93 ^ab^

^a,b^ Mean values in the same column followed by different letters are significantly different (*p* < 0.05). Control group (CT): without sodium nitrite and plasma treatment; NT: with 0.005% sodium nitrite; PT15: with plasma treatment for 15 min; PT30: with plasma treatment for 30 min; PT45: with plasma treatment for 45 min.

## References

[1] Brar S.K., Belkacemi K., Gassara F., Kouassi A.P. (2016). Green Alternatives to Nitrates and Nitrites in Meat-based Products-A Review. Crit. Rev. Food Sci. Nutr..

[2] Alahakoon A.U., Jayasena D.D., Ramachandra S., Jo C. (2015). Alternatives to nitrite in processed meat: Up to date. Trends Food Sci. Technol..

[3] Herrmann S.S., Granby K., Duedahl-Olesen L. (2015). Formation and mitigation of N-nitrosamines in nitrite preserved cooked sausages. Food Chem..

[4] Flores M., Toldra F. (2021). Chemistry, safety, and regulatory considerations in the use of nitrite and nitrate from natural origin in meat products—Invited review. Meat Sci..

[5] Pegg R.B., Honikel K.O. (2008). Principles of Curing. Handbook of Fermented Meat and Poultry.

[6] Riel G., Boulaaba A., Popp J., Klein G. (2017). Effects of parsley extract powder as an alternative for the direct addition of sodium nitrite in the production of mortadella-type sausages—Impact on microbiological, physicochemical and sensory aspects. Meat Sci..

[7] Sebranek J.G., Bacus J.N. (2007). Cured meat products without direct addition of nitrate or nitrite: What are the issues?. Meat Sci..

[8] Ozaki M.M., Munekata P.E., Jacinto-Valderrama R.A., Efraim P., Pateiro M., Lorenzo J.M., Pollonio M.A.R. (2021). Beetroot and radish powders as natural nitrite source for fermented dry sausages. Meat Sci..

[9] Ferysiuk K., Wojciak K.M. (2020). Reduction of Nitrite in Meat Products through the Application of Various Plant-Based Ingredients. Antioxidants.

[10] Zhu Y., Li C., Cui H., Lin L. (2020). Feasibility of cold plasma for the control of biofilms in food industry. Trends Food Sci. Technol..

[11] Pankaj S.K., Wan Z., Keener K.M. (2018). Effects of Cold Plasma on Food Quality: A Review. Foods.

[12] Oehmigen K., Hähnel M., Brandenburg R., Wilke C., Weltmann K.D., von Woedtke T. (2010). The Role of Acidification for Antimicrobial Activity of Atmospheric Pressure Plasma in Liquids. Plasma Process. Polym..

[13] Jung S., Lee C.W., Lee J., Yong H.I., Yum S.J., Jeong H.G., Jo C. (2017). Increase in nitrite content and functionality of ethanolic extracts of Perilla frutescens following treatment with atmospheric pressure plasma. Food Chem..

[14] Luo J., Yan W., Nasiru M.M., Zhuang H., Zhou G., Zhang J. (2019). Evaluation of physicochemical properties and volatile compounds of Chinese dried pork loin curing with plasma-treated water brine. Sci. Rep..

[15] Jung S., Kim H.J., Park S., Yong H.I., Choe J.H., Jeon H.-J., Choe W., Jo C. (2015). The use of atmospheric pressure plasma-treated water as a source of nitrite for emulsion-type sausage. Meat Sci..

[16] Lee J., Jo K., Lim Y., Jeon H.J., Choe J.H., Jo C., Jung S. (2018). The use of atmospheric pressure plasma as a curing process for canned ground ham. Food Chem..

[17] Yong H.I., Lee S.H., Kim S.Y., Park S., Park J., Choe W., Jo C. (2019). Color development, physiochemical properties, and microbiological safety of pork jerky processed with atmospheric pressure plasma. Innov. Food Sci. Emerg. Technol..

[18] Jo K., Lee J., Lee S., Lim Y., Choi Y.S., Jo C., Jung S. (2020). Curing of ground ham by remote infusion of atmospheric non-thermal plasma. Food Chem..

[19] Association of Official Analytical Chemists (1990). Nitrites in Cured Meat Colorimetric Method 973.31 Offical Methods of Analysis.

[20] Bao Y., Ertbjerg P. (2015). Relationship between oxygen concentration, shear force and protein oxidation in modified atmosphere packaged pork. Meat Sci..

[21] Oz F., Seyyar E. (2016). Formation of Heterocyclic Aromatic Amines and Migration Level of Bisphenol-A in Sous-Vide-Cooked Trout Fillets at Different Cooking Temperatures and Cooking Levels. J. Agric. Food Chem..

[22] Xiao X., Hou C., Zhang D., Li X., Ren C., Ijaz M., Hussain Z., Liu D. (2020). Effect of pre- and post-rigor on texture, flavor, heterocyclic aromatic amines and sensory evaluation of roasted lamb. Meat Sci..

[23] Liu H., Wang Z., Zhang D., Shen Q., Pan T., Hui T., Ma J. (2019). Characterization of Key Aroma Compounds in Beijing Roasted Duck by Gas Chromatography-Olfactometry-Mass Spectrometry, Odor-Activity Values, and Aroma-Recombination Experiments. J. Agric. Food Chem..

[24] National Institute of Standard and Technology Chemistry WebBook SRD 69[EB/OL]. https://webbook.nist.gov/chemistry/name-ser/.

[25] van Gemert L.J. (2011). Compilations of Odour Threshold Values in Air, Water and Other Media.

[26] Schieberle P., Hofmann T. (2011). Mapping the Combinatorial Code of Food Flavors by Means of Molecular Sensory Science Approach. Food Flavors.

[27] He W., Chung H.Y. (2019). Comparison between quantitative descriptive analysis and flash profile in profiling the sensory properties of commercial red sufu (Chinese fermented soybean curd). J. Sci. Food Agric..

[28] Jo K., Lee S., Yong H.I., Choi Y.-S., Jung S. (2020). Nitrite sources for cured meat products. LWT Food Sci. Technol..

[29] Zeghioud H., Nguyen-Tri P., Khezami L., Amrane A., Assadi A.A. (2020). Review on discharge Plasma for water treatment: Mechanism, reactor geometries, active species and combined processes. J. Water Process. Eng..

[30] Huang M., Wang J., Zhuang H., Yan W., Zhao J., Zhang J. (2019). Effect of in-package high voltage dielectric barrier discharge on microbiological, color and oxidation properties of pork in modified atmosphere packaging during storage. Meat Sci..

[31] Gavahian M., Chu Y.-H., Khaneghah A.M., Barba F.J., Misra N.N. (2018). A critical analysis of the cold plasma induced lipid oxidation in foods. Trends Food Sci. Technol..

[32] Jung S., Lee J., Lim Y., Choe W., Yong H.I., Jo C. (2017). Direct infusion of nitrite into meat batter by atmospheric pressure plasma treatment. Innov. Food Sci. Emerg. Technol..

[33] Kim B., Yun H., Jung S., Jung Y., Jung H., Choe W., Jo C. (2011). Effect of atmospheric pressure plasma on inactivation of pathogens inoculated onto bacon using two different gas compositions. Food Microbiol..

[34] Ursachi C.T., Pera-Crian S., Munteanu F.D. (2020). Strategies to Improve Meat Products’ Quality. Foods.

[35] Shahidi F., Pegg R.B. (1994). Hexanal as an Indicator of the Flavor Deterioration of Meat and Meat Products. Lipids in Food Flavors.

[36] Hospital X.F., Carballo J., Fernández M., Arnau J., Gratacós M., Hierro E. (2015). Technological implications of reducing nitrate and nitrite levels in dry-fermented sausages: Typical microbiota, residual nitrate and nitrite and volatile profile. Food Control..

[37] Perea-Sanz L., Lopez-Diez J.J., Belloch C., Flores M. (2020). Counteracting the effect of reducing nitrate/nitrite levels on dry fermented sausage aroma by Debaryomyces hansenii inoculation. Meat Sci..

[38] Liu H., Garrett T.J., Su Z., Khoo C., Gu L. (2017). UHPLC-Q-Orbitrap-HRMS-based global metabolomics reveal metabolome modifications in plasma of young women after cranberry juice consumption. J. Nutr. Biochem..

[39] Olatunde O.O., Shiekh K.A., Benjakul S. (2021). Pros and cons of cold plasma technology as an alternative non-thermal processing technology in seafood industry. Trends Food Sci. Technol..

[40] Watkins P.J., Jaborek J.R., Teng F., Day L., Castada H.Z., Baringer S., Wick M. (2021). Branched chain fatty acids in the flavour of sheep and goat milk and meat: A review. Small Rumin. Res..

[41] Giannoglou M., Dimitrakellis P., Efthimiadou A., Gogolides Ε., Katsaros G. (2020). Comparative Study on the Effect of Cold Atmospheric Plasma, Ozonation, Pulsed Electromagnetic Fields and High-Pressure Technologies on Sea Bream Fillet Quality Indices and Shelf Life. Food Eng. Rev..

